# Small interference RNA profiling reveals the essential role of human membrane trafficking genes in mediating the infectious entry of dengue virus

**DOI:** 10.1186/1743-422X-7-24

**Published:** 2010-02-01

**Authors:** Firzan Ang, Andrew Phui Yew Wong, Mary Mah-Lee Ng, Justin Jang Hann Chu

**Affiliations:** 1 Department of Microbiology, Yong Loo Lin School of Medicine, National University Health System, 5 Science Drive 2, National University of Singapore, Singapore 117597

## Abstract

**Background:**

Dengue virus (DENV) is the causative agent of Dengue fever and the life-threatening Dengue Haemorrhagic fever or Dengue shock syndrome. In the absence of anti-viral agents or vaccine, there is an urgent need to develop an effective anti-viral strategy against this medically important viral pathogen. The initial interplay between DENV and the host cells may represent one of the potential anti-viral targeting sites. Currently the involvements of human membrane trafficking host genes or factors that mediate the infectious cellular entry of dengue virus are not well defined.

**Results:**

In this study, we have used a targeted small interfering RNA (siRNA) library to identify and profile key cellular genes involved in processes of endocytosis, cytoskeletal dynamics and endosome trafficking that are important and essential for DENV infection. The infectious entry of DENV into Huh7 cells was shown to be potently inhibited by siRNAs targeting genes associated with clathrin-mediated endocytosis. The important role of clathrin-mediated endocytosis was confirmed by the expression of well-characterized dominant-negative mutants of genes in this pathway and by using the clathrin endocytosis inhibitor chlorpromazine. Furthermore, DENV infection was shown to be sensitive to the disruption of human genes in regulating the early to late endosomal trafficking as well as the endosomal acidic pH. The importance and involvement of both actin and microtubule dynamics in mediating the infectious entry of DENV was also revealed in this study.

**Conclusions:**

Together, the findings from this study have provided a detail profiling of the human membrane trafficking cellular genes and the mechanistic insight into the interplay of these host genes with DENV to initiate an infection, hence broadening our understanding on the entry pathway of this medically important viral pathogen. These data may also provide a new potential avenue for development of anti-viral strategies and treatment of DENV infection.

## Background

Many viruses have been identified for using the host endocytic pathways to mediate their infectious entry into host cells. These pathways include clathrin-mediated endocytosis, uptake via caveolae, macropinocytosis, phagocytosis, and other pathways that presently are poorly characterized [1]. Upon internalization into cells, some of these viruses are able to fuse with different cellular membranes or compartments and release their viral genome resulting in progressive virus infection [2]. The heavy reliance of the host membrane trafficking processes for virus entry process has also added advantages of allowing the virus to acquire a specific location within the cell for successful replication as well as to prevent the viruses from being recognized by the immune system [3]. Some viruses may require local cues such as low pH present in endocytic membrane vesicles to undergo penetration and to release their viral genome release into cells for replication [2]. Furthermore, the host cytoskeleton network such as actin filaments and the microtubule network may also be involved in the intracellular trafficking of the virus after being endocytosed [4]. The entry event is often a major determinant of virus tropism and pathogenesis [5]. Understanding the early event of virus replication cycle will provide opportunities to develop strategies to block this initial but crucial interaction.

Dengue virus (DENV) is a positive sense, single-stranded RNA virus belonging to the *Flavivirus *genus of the *Flaviviridae *family. DENV is commonly found in tropical regions globally and especially in urbanized cities. The *Flaviviridae *family of viruses which also includes, Japanese encephalitis virus, West Nile virus, yellow fever virus as well as the tick-borne encephalitis virus, is arthropod-borne and usually transmitted by infected ticks or mosquito vectors [6]. DENV is typically transmitted by two species of mosquitoes: *Aedes albopictus *and *Aedes aegypti*, which commonly breed in tropical parts of the world. DENV consists of 4 distinct serotypes (serotypes 1 through 4) and causes a wide range of diseases, starting with febrile Dengue fever (DF) to potentially fatal Dengue hemorrhagic fever (DHF)/Dengue shock syndrome (DSS). DENV causes an estimated 100 million infections annually with an increasing trend of many more countries becoming hyper-endemic for all 4 serotypes [7]. DF is characterized by fever, myalgia, arthralgia, headache, rash, and retro-orbital pain, however the disease is self-limiting. Symptoms of DHF/DSS on the other hand include thrombocytopenia, hemorrhage and increased vascular permeability ("plasma leakage"). DHF/DSS are potentially fatal if left untreated [8]. Despite the seriousness of DENV infection, there is currently no vaccine or anti-viral drugs available. For these reasons, a better understanding of the mechanism of infection by DENV is necessary to aid in the development of therapeutic strategies.

Dengue virus infection begins with attachment of virus particles onto host surface receptors followed by subsequent entry into the cell. It has been widely accepted that DENV enters permissive cells via receptor-mediated endocytosis and as such, a list of candidate receptors have been identified, some of which are cell-type specific. These cellular receptors include; heparan sulphate [9-12], heat shock protein (Hsp) 70 and Hsp 90 [13], GRP78/Bip [14], CD14 [15], a 37-kDa/67-kDa high affinity laminin receptor [16], dendritic cell (DC)-specific intracellular adhesion molecule 3 (ICAM-3)-grabbing non-integrin (DC-SIGN) [17-19] and liver/lymph node-specific ICAM-3-grabbing non-integrin [19]. Following internalization, the virus particles are postulated to uncoat within the endosomes with acidification, the envelope glycoprotein of DENV will undergo irreversible trimerization and resulting in endosomal fusion hence releasing the viral RNA into the host cytoplasm for replication [20].

Although previous studies have attempted to decipher the entry process of DENV as well as other mosquito-borne flaviviruses into host cells [21-25], little is currently known about the specific cellular genes or host factors that are involved in mediating the infectious entry of DENV into human cells. In the current studies, we have assessed an array of small interfering RNAs (siRNAs) libraries that specifically target human genes important for endocytosis processes, trafficking of membrane vesicles, actin polymerization and cytoskeleton rearrangement to determine the cellular genes or factors that facilitate the infectious entry pathway of DENV. Interestingly, we are able to show that the knockdown of human genes associated with clathrin-mediated endocytosis can efficiently block DENV infection. The essential involvement of clathrin-mediated endocytosis in DENV entry into cells was confirmed by the expression of dominant-negative mutants and drug inhibitors to perturbate this uptake pathway. In addition, we have also identified cellular factors responsible for vesicle trafficking and maturation, signal transduction and actin polymerization that are essential for the infectious entry process of DENV.

## Results

### Optimization of siRNA screening platform for DENV infection

**Figure 1:**
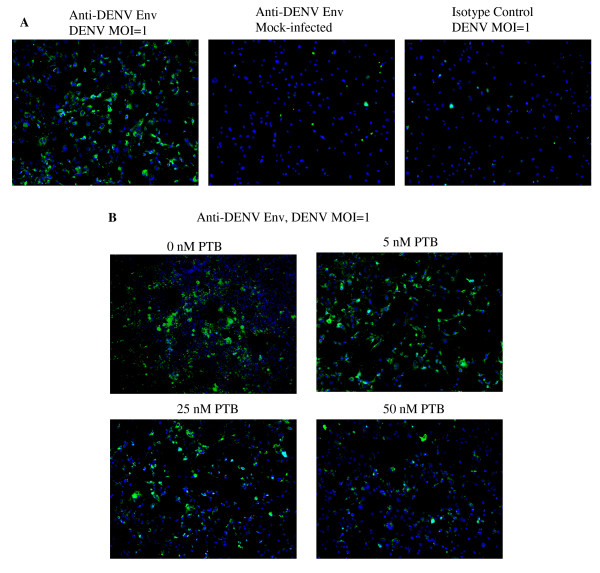
**Development of an image-based DENV detection assay for siRNA screening in Huh7 cells**. (A) Detection of DENV infection in Huh 7 cells using immunofluorescence assay with antibody specific for the viral E protein and the cell nuclei are stained with DAPI. (B) Dosage-dependent inhibition of DENV infection is observed in Huh7 cells that are reverse-transfected with different concentrations of siRNA against PTB.

In this study, a screening platform for DENV replication was adapted from [26] and optimized to detect siRNA capable of interfering with the different step(s) of the DENV replication cycle through their direct effects on cellular factors that participate in these viral processes. The immunofluorescence-based screening assay is based on the detection of DENV envelope protein in DENV-infected cell monolayers (Figure 1A). We first evaluated the ability of the assay to quantitatively detect inhibition of DENV infection by using siRNA for specific knock-down of polypyrimidine tract-binding protein (PTB), which is known to inhibit the viral RNA synthesis of DENV [27]. Different concentrations of siRNA that target PTB were first reverse-transfected into Huh7 cells cultured in a 384-well plate and followed by DENV infection at a multiplicity of infection (MOI) of 1. Three days after infection, cells were fixed and stained for viral envelope (E) protein. By using an automated image-capturing microscope, the cytoplasmic green fluorescence (DENV E protein) and nuclear blue fluorescence acquired from four selected fields in each well were imaged and the average number of DENV-infected cells was then determined by automated data analysis. As shown in Figure 1B, the reduction in the number of cells staining positively for DENV E protein with increasing concentrations of siRNA specific for PTB when compared with cells reverse-transfected with the scrambled sequence of PTB siRNA or mock-transfected cells. This result was consistent with the previous report on the inhibitory activity of DENV replication upon gene silencing of PTB [27]. In addition, to ensure that the screening assay has minimal signal variation and a consistently high signal-to-background ratio, we have also determined the Z' factor of the screening assay [28] based on data collected from 120 wells of DENV-infected cells reverse-transfected with 50 μM of siRNA against PTB and data collected from another 120 wells of the same 384-well plate that were reverse-transfected with 50 μM of siRNA with scrambled sequence of PTB. A Z' factor of 0.70 was consistently observed, demonstrating the reliability and robustness of this assay. With the optimization of this siRNA screening platform for DENV infection, we have employed this screening platform as a tool for rapid discovery of cellular factors that is essential for mediating the infectious entry process of DENV into cells.

### siRNA profiling of human membrane trafficking genes required for DENV infection

**Figure 2:**
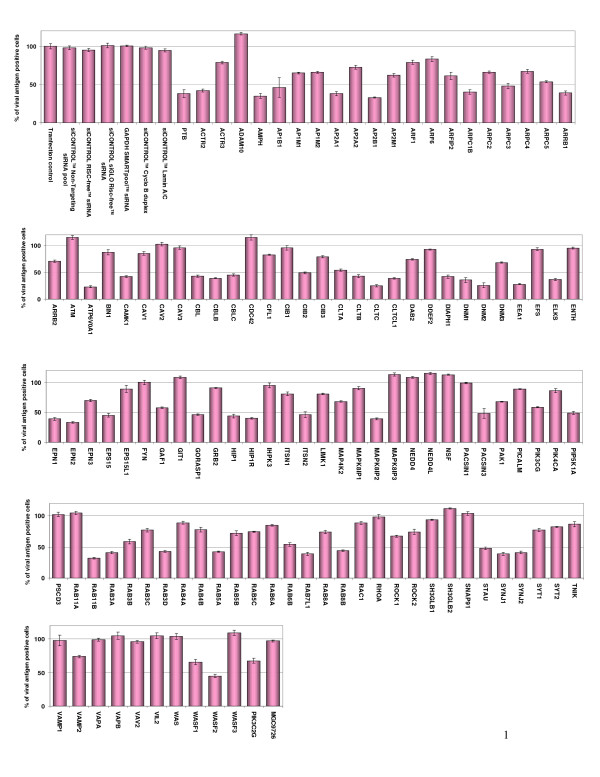
**Identification of human genes that is important in endocytosis, vesicle trafficking and signaling as well as cytoskeleton rearrangement on DENV infection using siRNA screening platform**. Huh7 cells were reverse-transfected with the panel of 119 siRNAs and, after 2 days, the cells were infected with DENV at an MOI of 1. After 48 hrs post-infection, the DENV infected cells were processed for immunofluorescence staining, auto-image capturing and data analysis. The data is expressed as the percentage of antigen-positive cells and the results are shown from three independent sets of experiments. To establish a baseline of infection and transfection efficiency, cells are treansfected with a control set (top left) of siRNAs and/or transfection reagent, which include transfection lipids alone or together with "non-targeting" siRNA pool, RISC-free siRNA, siGLO (fluorescently labeled) RISC free non-specific siRNA or siRNAs targeting glyceraldehyde-3-phosphate dehydrogenase (GAPDH), Cyclo B duplex and lamin A/C. Together with these controls permitted monitoring of transfection efficiency and cytotoxicity. Accession numbers and a brief description of the role of each gene are provided in Additional File 1.

An array of 119 siRNA pools targeting genes known to be directly or indirectly involved in regulating the different endocytosis pathways (clathrin, caveolae, macropinocytosis, etc), polymerization of actin & cytoskeleton rearrangement, and vesicle/cargo trafficking was used to identify host genes necessary for the infectious entry of DENV. A list of the targeted human genes and a brief description of the reported functional role for each of the genes are provided in Additional File 1. We have utilized a less than 50% of viral antigen positive cells as the siRNA-induced effect and criterion that suppressed DENV infection. The list of human genes that have an inhibitory effect on DENV infection are obtained using the screening platform and the results are shown in Figure 2 and further classified based on their functional roles as indicated in Additional File 2.

Interestingly, the siRNAs that gave the strongest inhibition of DENV infection were those that targeted genes involved in the process of endocytosis (Figure 2 and Additional File 2), these included clathrin heavy chain (CLTC; 75% inhibition), the subunit of the clathrin-associated adapter protein complex 2 (AP2A1; 62%, AP2B1; 67%), endocytic accessory protein for clathrin-coated pit formation (EPN2; 67%), dynamin 2 (DNM2; 74%), Rab5 (62%), Rab11B (68%) and Rab7 (61%). The clathrin heavy and light chains are intricately braided together to form the triskelion coat that facilitate the formation of clathrin coated pits for endocytosis [29]. AP2A1 and AP2B1 are the subunits of the clathrin-associated adaptor protein complex 2 that regulate the formation of clathrin-coated pits as well as linking clathrin to cellular receptors in endocytic vesicles [30]. Epsin 2 (EPN2) plays important role in recruiting of clathrin molecules to membranes and promotes its polymerization to mediate endocytosis [31]. Dynamin 2 is necessary for the regulation of actin-membrane interaction for pinching off of clathrin coated pits from the plasma membrane [32]. The Rab proteins (Rab 5, 7 and 11b) associate with the endocytic membrane component with critical role in the endocytosis process and the formation of early endosomes [33]. In addition, siRNAs that target genes encoding for proteins that regulate the trafficking and maturation of endocytic vesicles were also strong inhibitors of DENV infection (Figure 2 and Additional File 2). These human genes include early endosome antigen 1 (EEA1; 72%), Rab6 interacting protein 2 (ELKS; 63%) and ATPase (ATP6V0A1; 77%). Both EEA1 and ELKS are required for the intracellular trafficking of endosomes from the plasma membrane [34,35]. ATP6V0A1, a subunit of the ATP-driven vacuolar proton pump that is associated with clathrin-coated vesicles/endosomes and is essential for the acidification processes within these vesicles [36]. Furthermore, siRNAs that target kinases (MAPK8IP2; 61%) and kinase adaptor proteins (CBLB; 61%) that are involved in the signal transduction processes of viral entry [37] was also noted to reduce DENV infection. Lastly, the siRNAs knock-down of human genes that are essential in regulating actin polymerization also have an inhibitory effect on DENV infection (Figure 2 and Additional File 2). These include genes that contribute to the subunit component of actin related 2/3 complex that is implicated in actin assembly, actin cytoskeletal remodeling as well as cellular signaling via actin.

In these experiments, we have also included a number of transfection and cellular controls (as indicated in Figure 2) and it was observed that generally there was minimal cytoxicity in the siRNA reverse-transfected cells with DENV infection. The only exception was that cells reverse-transfected with the cytotoxic siRNA control (*Kif11*) showed more than 80% cell loss. All other siRNAs did not affect cell viability sufficiently to contribute to the outcome of the screen.

To further validate the findings obtained from the initial screen, we have selected these genes (CLTC, AP2B1, DNM2, ARRB1, ATP6V0A1 & ARPC1B) for re-confirming their inhibitory effects on DENV infection. Different concentrations of each specific siRNA directed against the respective genes are reversed transfected into Huh 7 cells and further subjected to DENV infection. In addition, Western blotting was carried out to confirm the siRNA treatment has effectively suppressed the expression of the targeted genes. These experiments will also ensure that the inhibitory effect on DENV infection is not due to the off-target gene effect of the siRNA treatment.

**Figure 3:**
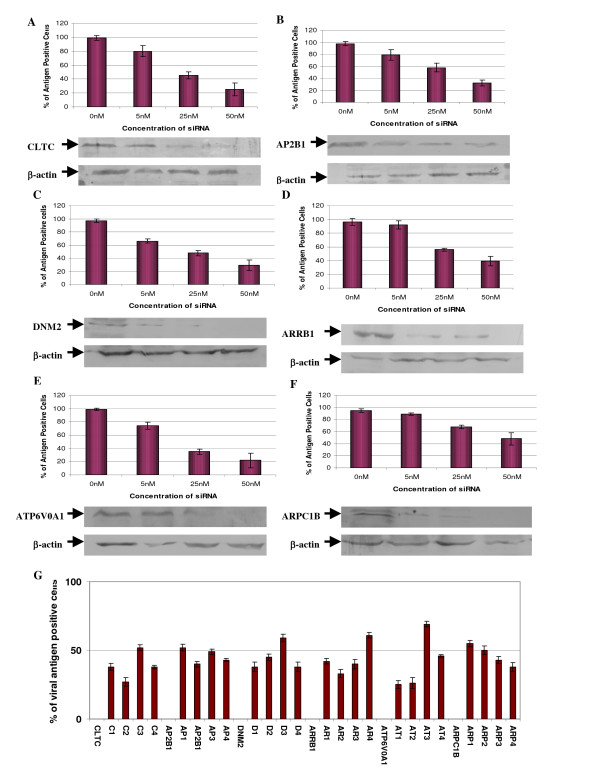
**Confirmation of siRNA suppression of host protein expression and reduction of DENV infection**. Gene specific siRNA against (A) CLTC, (B) AP2B1, (C) DNM2, (D) ARRB1, (E) ATP6V0A1 and (F) ARPC1B were reverse-transfected into Huh7 cells at different concentrations (0 nM to 50 nM) and subjected to DENV infection. Dosage dependent inhibition of DENV infection can be observed for these selected genes. At the same time, Western blots were performed after treatment with siRNAs to ensure the knockdown of the specific protein expression. Dosage-dependent reduction of protein expression is also observed for the indicated genes corresponding to the concentrations of the transfected siRNA. The blots are also re-probed with β-actin-specific antibody which served as a gel-loading control (lower panels). (G) Deconvolution of siRNA smartpools that reduced infectious entry of DENV. The experiments shown were repeated with the deconvoluted siRNA sequences (4 individual siRNA) from the Smartpool. The data were displayed for 3 independent experiments.

Reverse-transfection of Huh 7 cells with siRNAs targeting CLTC, AP2B1, DNM2, ARRB1, ATP6V0A1 and ARPC1B showed dosage dependent reductions in the levels of the respective proteins when compared to the levels in the mock-transfected cells (Figure 3). At the concentration of 25 nM of the transfected siRNA for the respective proteins, more than 65% reduction (as measured by densitometry) can be observed when compared to the mock-transfected samples. Similarly, with the knock-down of the respective genes (CLTC, AP2B1, DNM2, ARRB1, ATP6V0A1 and ARPC1B) with the different concentrations of the siRNAs, a dosage dependent inhibition of DENV infection can be observed (Figure 3A-F). The knock-down of ATP6V0A1 and CLTC (at concentration of 50 nM) produced the strongest inhibition of DENV infection. siRNA smart pool-based deconvolution assays targeting CLTC, AP2B1, DNM2, ARRB1, ATP6V0A1 and ARPC1B were also performed to ensure that inhibitory effects on DENV infection observed in the primary screen was specific and not due to off-target gene effects of the siRNA primary screen. 30 nM of each specific individual siRNA of the smart pool (4 specific siRNAs) directed against each of the respective genes were reverse transfected into RD cells and subsequently subjected to DENV infection. It can be observed that at least one of the four siRNAs directed against each specific gene (CLTC, AP2B1, DNM2, ARRB1, ATP6V0A1 and ARPC1B) can result in more than 50% inhibition of DENV infection (Figure 3G) hence indicating the specificity of these genes in mediating DENV infection.

Together, these data may indicate a strong correlation between the impacts of each transfected siRNA on DENV infection upon the suppression of protein expression. Furthermore, it is highly suggestive that the endocytosis of dengue virus into cells is dependent on clathrin, actin cytoskeletal dynamics as well as endosome trafficking and acidification.

### Infectious entry of DENV into cells involved clathrin-mediated endocytosis

**Figure 4:**
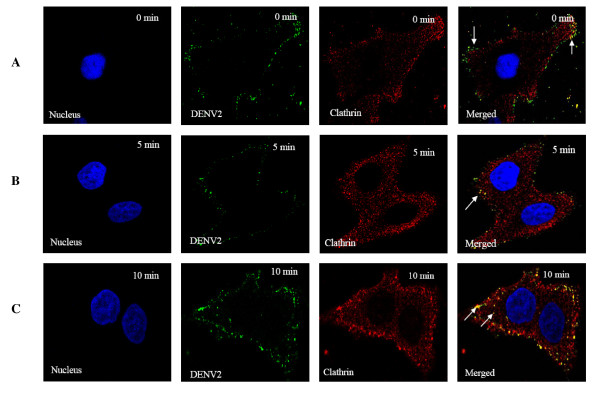
**Bio-imaging analysis of the interaction of clathrin molecules with DENV**. DENV were stained green with anti-DENV E protein antibody conjugated to FITC, host clathrin stained red with anti-clathrin antibody conjugated to Texas Red (TR) and host nuclei stained blue with DAPI. (A) Attachment of DENV on the cell surface can be observed at 0 min p.i (arrow) with few co-localizations between DENV and clathrin molecules (arrows) (B) Obvious co-localization is observed between internalized DENV and clathrin by 5 minutes p.i (arrow). (C) Strong co-localization signals are observed between DENV2 and clathrin by 10 minutes p.i (arrows).

To further characterize the involvement of clathrin in mediating the infectious entry of DENV, double-labeled immunofluorescence assay was performed to track the entry process and cellular localization of DENV within cells at the appropriate times post-infection. At 0 min after cells were warmed to 37°C, DENV particles (green) were observed predominantly at the plasma membrane of the cells and co-localization of virus particles with clathrin (possibly clathrin-coated pits, arrows) were noted too (Figure 4A). Within 5 to 10 min post-infection, strong co-localization of DENV particles and clathrin (arrows) within the cytoplasm were observed (Figure 4B and 4C). The co-localization of clathrin coated vesicles with DENV was further verified by 3D spectral confocal imaging. In particular, strong co-localization was observed between DENV2-infected Huh7 cells and clathrin molecules (Additional File 3). Together, these data suggest the involvement of clathrin in the endocytosis of the DENV particles.

**Figure 5:**
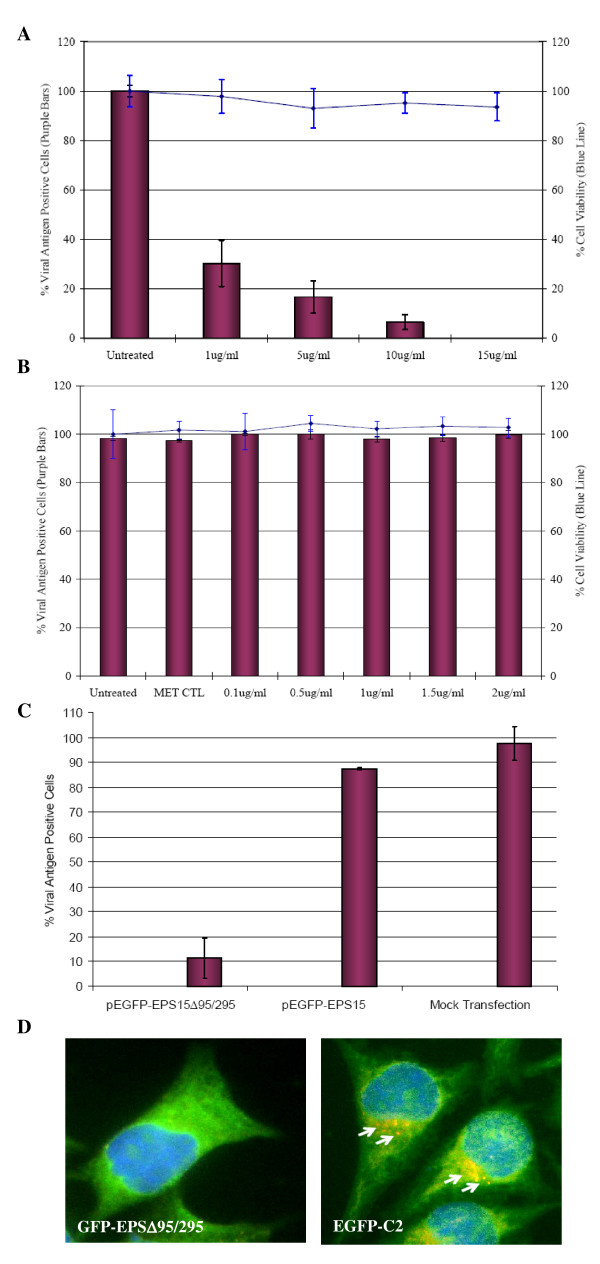
**Clathrin-mediated endocytosis of DENV into Huh7 cells**. Huh7 cells treated with (A) chlorpromazine shows marked reduction in the infectious entry of DENV, whereas (B) filipin does not significantly inhibit virus entry. The solvent (MET, methanol) control for filipin treatment is also included. Minimal cytotoxicity was observed for the concentration range of chlorpromazine and filipin used in this assay. The average of three independent experiments is shown. (C) Inhibition of DENV entry into Huh7 cells expressing EPS15 dominant-negative mutant protein. The infectious entry of DENV is significantly inhibited in Huh7 cells transfected with GFP-EPSΔ95/295 when compared to mock-transfected or pEGFP transfected cells. The number of viral E antigen-positive cells in relation to the total cell population is expressed as a percentage of viral antigen-positive cells. The average of three independent experiments is shown. (D) DENV (stained with TR failed to infect GFP-EΔ95/295 expressing cell. In contrast, internalized DENV particles (arrows) are observed within cells expressing the negative control plasmid (EGFP-C2) expressing GFP.

To affirm the role of clathrin-mediated endocytosis in DENV infection, Huh 7 cells were pretreated with drugs that selectively inhibit clathrin-dependent endocytosis (chlorpromazine) and caveola-dependent endocytosis (filipin, which disrupts the cholesterol-rich caveola-containing membrane microdomain) and then challenged with the virus. Pretreatments of Huh7 cells with chlorpromazine for 2 h before DENV infection significantly reduced the number of infected cells in a dosage dependent manner (Figure 5A). In contrast, filipin had no significant effect on DENV infection regardless of the concentrations of the drug added to the cells (Figure 5B). Minimal cytotoxicity was observed for the concentrations of the drugs used in this part of the experiments.

### Dominant negative EPS15 mutants inhibit DENV entry into cells

Molecular inhibitors in the form of dominant-negative mutants were also used to further confirm the role of clathrin-mediated endocytosis in the infectious entry of DENV. The use of dominant-negative mutants may provide an alternative way to analyze the specific function of defined pathways within the cells. Previous study by Benmerah *et al *has shown that EPS15, a protein that binds to the AP-2 adapter, is required for internalization through clathrin-coated pits [38]. However, the deletion of the EH domain of EPS15 produced a dominant-negative mutant of the protein that abolished functional clathrin-coated pit formation and inhibits clathrin-mediated endocytosis [39]. In this part of the study, Huh7 cells were first transfected with either GFP-EPSΔ95/295 plasmid (dominant-negative mutant of EPS15) or EGFP-C2 plasmid (coding for green fluorescent protein [GFP] as an internal control) as previously described by [39]. The transfected cells were then assayed for their capacity to internalize FITC-conjugated transferrin (specific cellular marker for clathrin-mediated endocytosis). The internalization of FITC-transferrin was severely impaired in cells expressing GFP-EPSΔ95/295, but not in cells that express GFP (data not shown).

As shown in Figure 5C, the over-expression of GFP-EPSΔ95/295 greatly reduced the level of DENV infection compared to that of the GFP control and the mock-transfection control (77% and 79% reduction, respectively, *P *< 0.001) of Huh7 cells. It was noted that the effect of the dominant-negative gene was specific and not due to the GFP tag, since the levels of infection of cells expressing GFP alone were not much different from the levels in non-transfected cells. Minimal cellular cytotoxicity was also observed for the transfected cells (data not shown). Furthermore, GFP-EPSΔ95/295 or GFP expressing cells were also incubated with DENV at 37°C for 30 min and processed for immunofluorescence staining and microscopic imaging. DENV particles failed to enter the GFP-EPSΔ95/295 expressing cells (Figure 5D). In contrast, Figure 5D shows the internalization of DENV (arrows, speckled staining) within the cytoplasm of GFP-expressing cells. These results together provide strong evidence that DENV entry into cells takes place through clathrin-mediated endocytosis.

### siRNA knockdown of clathrin heavy chain inhibits infectious entry of all four DENV serotypes

**Figure 6:**
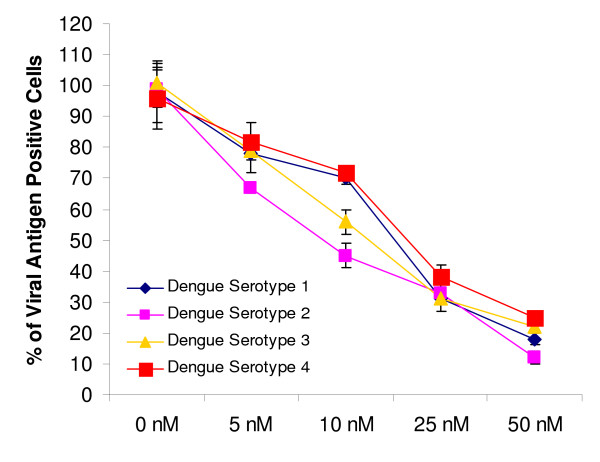
**siRNA knockdown of clathrin heavy chain inhibits all DENV serotypes infection**. Huh7 cells were reverse-transfected with different concentrations of clathrin heavy chain (CLTC) specific siRNA pool have resulted in dosage-dependent reduction of all DENV serotypes infection. The average of three independent experiments is shown.

To determine whether the different serotypes of DENV (DENV 1 to 4) utilized clathrin-mediated endocytosis to gain entry into Huh7 cells, cells were reverse-transfected with different concentration of siRNAs that target clathrin heavy chain (CLTC) and subjected to DENV 1 to 4 infection (MOI of 1). As shown in Figure 6, dosage dependent inhibition of DENV infection was observed for all serotypes. In comparison, there was minimal inhibition of DENV infection (all serotypes) for Huh7 cells transfected with scrambled sequence CLTC and mock-transfected cells (data not shown). These data strongly suggest that clathrin-mediated endocytosis is indeed responsible for the entry process of the different DENV serotypes.

### Endocytic trafficking of internalized DENV particles within cells

**Figure 7:**
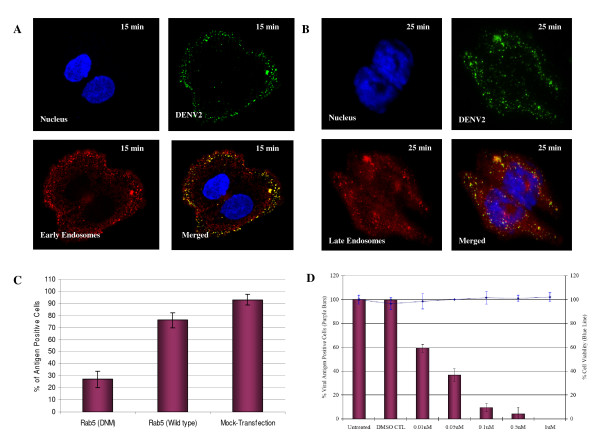
**Endosomal trafficking and low pH dependency is required to mediate infectious entry of DENV into cells**. (A) Immunoflourescence analysis reveals co-localization of DENV particles with early endocytic vesicles. Anti-EEA1 was used to stain the early endosomes at 15 min p.i. while DENV particles were stained by anti-DENV E protein antibody conjugated with FITC. (B) By 25 min p.i., majority of the DENV particles are found in association with late endocytic vesicles as shown by confocal imaging. Lysotracker (specific stain for late endosomes) was used to stain the late endosomes within Huh7 cells. Perinuclear distribution pattern of the DENV-associated late endsomes is observed too. (C) Infectious entry of DENV is strongly inhibited in Huh7 cells transfected with dominant negative mutant of Rab5 (DNM) as compared to cells transfected with wild-type Rab5 or mock-transfected cells. (D) Bafilomycin A1 pretreatment of Huh7 cells significantly reduce DENV infection in a dosage-dependent manner. Minimal cytotoxicity is observed for the concentration range of bafilomycin A1 used in this assay. The solvent (DMSO) control was also included in this set of experiment. The average of three independent experiments is shown.

Next, to further substantiate the role for clathrin-mediated endocytosis in DENV infection, the importance of early endosome trafficking of internalized DENV particles was first visualized by microscopic assay. Within 15 min post-infection, a double-labeled immunofluorescence assay with anti-DENV envelope protein and anti-EEA1 antibodies showed colocalization, suggesting that the virus particles were translocated to the early endosomes after clathrin-mediated endocytosis (Figure 7A). At this time point, most of the virus-containing endosomes were distributed closer to the cell periphery (Figure 7A). By 25 min post-infection, DENV particles are found mainly in vesicles (Figure 7B) that were stained with Lysotracker (Molecular Probes), suggesting that DENV were localized to the late endosomes by this time point. The fluorescent staining was more intense at the perinuclear region. The involvement of endosomal trafficking of internalized DENV particles was further evaluated by using molecular inhibitor of dominant negative mutant of Rab5 (a protein required for early endosome formation and trafficking) [40]. Huh7 cells were first transfected with GFP-tagged forms of Rab5 (dominant negative and wild type) and subjected to DENV infection at MOI of 1. The over-expression of wild-type Rab5 reduced DENV infection levels to a small but significant extent (15%; *P *< 0.001; Figure 7C). In fact, this observation was consistent with the previous reports that the over-expression of Rab5 can alter endocytosis by increasing receptor (with attached virus) recycling back to the cell surface [41]. In contrast, the dominant-negative Rab5 mutant had a more-dramatic effect, reducing the levels of DENV infection by 65% (*P *< 0.001; Figure 7C). The data obtained so far thus suggest the trafficking of internalized DENV particles require early-to-late endosome maturation to infect cells, instead of exiting the endosomal pathway soon after early endosome formation.

The pH-dependent requirement for infectious entry of DENV was earlier indicated by the siRNA knockdown of VTPase (ATP6V0A1) in this study. This was further assessed by using a highly specific vacuolar H^+^-ATPase (VATPase) inhibitor - bafilomycin A [42]. As shown in Figure 7D, bafilomycin A caused a marked reduction of DENV infection in a dose-dependent manner. Possible cytotoxic effects of the drugs were also assessed by MTT assay and observation of morphological changes. Minimal cell toxicity was observed in drug-treated cells throughout the spectra of concentrations used in this experiment.

### Infectious entry of DENV is dependent on cytoskeletal network

**Figure 8:**
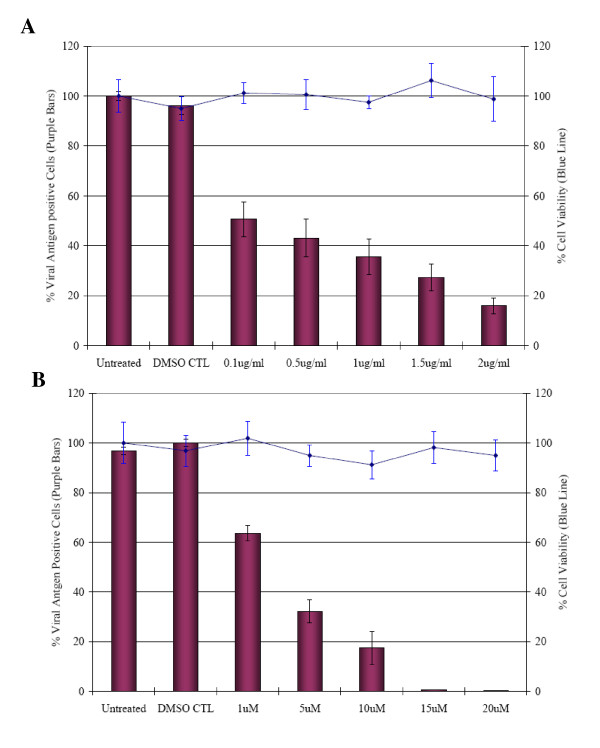
**Involvement of the cytoskeleton networks in the entry process of DENV virus**. (A) Cytochalasin D or (B) nocodazole-pretreated Huh7 cells is shown to inhibit the entry of DENV in a dosage-dependent manner. The percentage of viral antigen-positive cells is plotted against time. Minimal cytotoxicity was observed for the concentration range of the drugs used in this assay. The solvent (DMSO) control was also included in this set of experiment. The average of three independent experiments is shown.

The cytoskeleton also plays a dynamic role in endocytic trafficking and these processes can be inhibited at different stages by specific drugs (cytochalasin D and nocodazole). Cytochalasin D and nocodazole induced depolymerization of actin filaments and microtubules, respectively. Durrbach and co-workers [43] documented the sequential involvement of both actin filaments and the microtubule network in the trafficking pathway of ligands via clathrin-mediated endocytosis. Non-cytotoxic concentrations of cytochalasin D (0.1 to 2 μg/ml) and nocodazole (1 to 20 μM) were used to assay for DENV infection. Pretreatment of Huh7 cells with increasing concentrations of either cytochalasin D or nocodazole revealed a dose-dependent inhibition of DENV infection (Figure 8A and 8B).

## Discussion

Attachment of the virus to the cell surface followed by viral entry is the first step in a cascade of interactions between the virus and the target cell that is required for successful entry into the cell and initiation of infection. This step is an important determinant of tissue tropism and pathogenesis of virus infection; it thus represents a major target for antiviral host cell responses and the development of anti-viral strategies against virus infection.

Many viruses (enveloped or non-enveloped) depend on the host cell's endocytic pathways for entry [44]. They follow a multistep entry and uncoating process that allows them to move from the cell periphery to the perinuclear space. The interaction between the virus and the host cell is initiated with virus binding to attachment factors or receptors on the cell surface, followed by lateral movement of the virus-receptor complexes and the induction of signals that result in the endocytic internalization of the virus particle.

In general, endocytic trafficking involves vesicle-mediated flow and exchange of membrane components from the plasma membrane into the cell. Endocytic vesicles are formed by invagination of the plasma membrane, and in most cases, the initial destination of endocytic vesicles is the endosome. Subsequently, the uptaken specific cargo can be sorted and selected for trafficking to other intracellular compartments, including the multivesicular body (MVB) and lysosomal pathway [45]. As such, the endocytic trafficking pathway can be hijacked by viruses to mediate their infectious entry into host cells and facilitate their replication in specific cellular components.

Among the many challenges to understand the replication process of DENV is the need to identify exactly the endocytic mechanism that contribute to the infectious entry of DENV into susceptible cells. The application of RNA interference-based screens may offer an alternative route to identify the cellular proteins or components of endocytic pathways that mediate the infectious entry of DENV. Despite its recent discovery, the application of RNA interference has already profoundly enhanced the study of large-scale loss-of- functional gene analysis in a rapid and cost-effective manner. For this purpose, we have established and validated a RNA interference screening platform assay that allows identification of host proteins involved in the endocytic and membrane trafficking process that mediate the entry of DENV into cells.

In this study, clathrin-mediated endocytosis is identified as the main pathway of DENV internalisation, since knockdown of clathrin heavy chain and dynamin inhibits uptake by 70% or more (Figure 2 and Additional File 2). In addition, knockdown of several other endocytic proteins including epsin, syndapin, arrestin, subunits of clathrin adaptor protein complex, Rab5, Rab7 and Rab11B also significantly reduces the uptake of DENV into Huh7 cells (Figure 2 and Additional File 2). The formation of clathrin-coated vesicles are generated by the joint action of machinery that includes clathrin heavy and light chains, adaptor complexes (AP1 and AP2), membrane-bending proteins such as epsin, amphiphysin, syndapin and endophilin A, the proposed 'pinchase' dynamin, and many 'accessory' proteins (Rab GTPases) that regulates other endocytic proteins [32]. The complex interactions of these cellular proteins are believed to have important roles in controlling the endocytosis kinetics and possibly the origins and destinations of cargoes. Furthermore, the functional role of clathrin-mediated endocytosis in mediating DENV entry was independently confirmed by microscopic cellular localization analysis (Figure 4), chlorpromazine treatment of cells and by transfecting cells with a well-characterized dominant-negative mutant form of EPS15. Co-localization of DENV particles with clathrin molecules can be observed within the first 10 min of post-infection. Chlorpromazine is a well-known specific inhibitor of clathrin-mediated endocytosis [46]. EPS15 is an accessory factor that associates with AP2 complex and is essential for the formation of clathrin-coated pits at the plasma membrane [47]. The dominant-negative form of EPS15 has been shown to effectively blocks clathrin-mediated endocytosis but not other endocytosis processes by caveolae or macropinocytosis [39]. Both the drug treatment and the expression of dominant-negative EPS15 were effective inhibitors of DENV infection (Figure 5). Together, these different experimental approaches strongly indicate a key role for clathrin function in the entry of DENV into cells. In consistent with involvement of clathrin in mediating DENV entry into human hepatocyte cell line in this study, several other studies have also documented the functional role of clathrin in facilitating the infection of DENV in human cells and mosquito cells [21,22,48,49]. Several other members [bovine viral diarrhea virus [23]], Japanese encephalitis virus [24] and West Nile virus [25] and of the *Flaviviridae *family were also shown to enter cells via clathrin-mediated endocytosis. Therefore, it seems that clathrin-mediated endocytosis may be the generalized uptake process for many members of the *Flaviviridae *family.

The vacuolar protein sorting (VPS) pathway is known to be essential in endocytic trafficking of cargo proteins through early endosomes into late endosomes/MVBs and on to lysosomes as well as the recycling of proteins from early and late endosome to the Golgi [50,51]. Our primary siRNA screen has also indicated that targeting of the endocytic trafficking proteins and associated kinases, Rab5A, Rab7, Rab11b, ELKS, early endosome antigen 1 and MAPK8IP2, each resulted in significant decreases in DENV infection (Figure 2 and Additional File 2). Rab5 is required and directly controls the formation of early endosomes [40]. The dominant-negative mutant forms of Rab5 confirmed a need for early endosome formation for DENV infection. This observation was further confirmed by the co-localization of DENV particles with early endosomes within 15 min post-infection. The trafficking-maturation model of endosomes has suggested that endosomes act as transient independent carriers that progressively change in size and shape by homologous fusion and eventually mature into late endosomes and lysosomes [52,53]. This process was also observed in the trafficking of the internalized DENV from early endosomes to late endosomes as revealed by immunofluorescence assay (Figure 7).

It is also notable that the specific siRNA knockdown of vacuolar H^+^-ATPase6V0A1 (both primary and secondary siRNA assays) has resulted in drastic inhibition of DENV infection, hence indicating the essential requirement of acidification within endosomes for infectious entry of DENV. This was further confirmed by incubation of cells with vacuolar H^+^-ATPase (VATPase) inhibitor (bafilomycin) that caused a marked reduction in DENV infection (Figure 7D). This observation is consistent with a number of enveloped viruses that are currently considered to be pH dependent for the viral uncoating process along endocytic pathway [54]. For flaviviruses, it has been proposed that the structural domain II of the E protein require a pH-dependent fusion with the endocytic membranes to release the viral genome for replication. Mutational studies that disrupt the functional biology of this domain have shown a decrease in the replication and virulence of these flaviviruses [55]. A highly conserved amino acid cluster located at the tip of domain II has been proposed to be the internal fusion peptide. At the low pH of fusion, the flavivirus E protein on the surface of the virus undergo irreversible conformational changes to expose the fusion peptide for interaction with the target membrane [56].

In addition, the cytoskeleton (actin filaments and microtubules) also plays a dynamic role in endocytic trafficking, with both up- and down-regulation of actin or mictotubule polymerization is shown to affect the kinetics of endocytosis [57]. Actin filaments are required for the initial uptake of ligands via clathrin coated pits and subsequent degradative pathway, whereas microtubules are involved in maintaining the endosomal traffic between peripheral early and late endosomes [43,57]. Actin cytoskeleton is shown to be closely associated with clathrin-coated pits and that actin polymerization may be involved in moving endocytic vesicles into cytosol after they are pinched off from the plasma membrane [58]. The actin-binding molecular motor, myosin VI, was also recently shown to mediate clathrin endocytosis [59]. Disruption of actin filaments can have a dramatic effect on receptor-mediated endocytosis [57]. In this study, the siRNA knockdown of ACTR2, ARPC1B, ARPC3 (different subunits of the actin related 2/3 protein complex), ARRB1, DIAPH1, PIP5K1A and WASF2 genes that are important in actin polymerization have resulted in reduction of DENV infection. These proteins are known to be important in coupling clathrin-mediated endocytosis to actin. β-Arrestin (ARRB1) is involved in the desensitization of receptors by targeting them to clathrin-coated vesicles through a RhoA and actin-dependent mechanism [60]. WASF2 is an important down-stream effector of receptor-mediated signaling that triggers actin polymerization. WASF family members also play important roles late in clathrin-coated pit formation by coupling to the ARP2/3 actin-regulating complex and may act to move the vesicle through the cell [61]. Therefore, these results suggest a potential role of these genes in regulating DENV endocytosis after binding to putative cellular receptors. The involvement of actin in mediating DENV virus entry was further confirmed by treatment with cytochalasin D. Cytochalasin D, an actin-disrupting drug, specifically affects the actin cytoskeleton by preventing its proper polymerization into microfilaments and promoting microfilament disassembly [62]. Disruption of actin filament was shown to inhibit DENV infection in a dosage dependent manner (Figure 8A). Furthermore, we are able to show the functional role of microtubule in DENV infection. Treatment of cells with nocodazole (disrupts microtubules by binding to β-tubulin and preventing formation of one of the two interchain disulfide linkages, thus inhibiting microtubules dynamics) effectively inhibit DENV infection (Figure 8B). Microtubules are required for efficient transcytosis and for trafficking of early endosomes to late endosomes [57]. Therefore, it appears noteworthy that the endocytic pathway for DENV is closely associated with host cells cytoskeleton network.

This current work has highlighted the power of using specific subset siRNA libraries to identify important cellular genes and pathways that mediate the process of endocytosis and endocytic trafficking of DENV infection. Although previous RNA interference screening studies [63,64] that are carried out at the entire genomic level have also identified some similar genes involved in the entry mechanism of DENV but this current study has provided much in-depth analysis of human genes that are essential for the endocytosis as well as the endocytic trafficking of internalized DENV for productive infection. Understanding these processes may allow specific cellular pathways or molecular mechanisms to be targeted pharmacologically to inhibit the entry of DENV that uses the route for infection. Such an approach may offer the advantage that it will be more difficult for a virus to find a way around the block by mutation. Furthermore, the development of specific drugs, dominant negative gene mutants and RNAi therapeutic approach targeting virus entry can be effective in disease intervention of DENV or even other related pathogenic flaviviruses.

## Methods and materials

### Cell cultures and virus preparation

Huh7 and HepG2 cells (human hepatoma cell lines, America Type Culture Collection) were maintained in Dulbecco's modified Eagle's medium (DMEM; Gibco) containing 10% inactivated fetal calf serum (FCS). Low passage human isolate of DENV viruses (Serotype 1, 2, 3 & 4, Singapore isolates) was kindly provided by the Department of Pathology, Singapore General Hospital. DENV serotype 2 was used for all experiments in this study except for the experiments on the siRNA knockdown of the clathrin heavy chain whereby all four serotypes were assessed. C6/36 cells were used to propagate this virus throughout this study. In brief, confluent monolayers of C6/36 cells were infected with DENV viruses at a multiplicity of infection (MOI) of 10. At 4 days post-infection (p.i.), the supernatant was harvested by centrifugation at 5,000 rpm for 10 min. DENV viruses were then concentrated and partially purified by using a centrifugal filter device (Millipore, Bedford, Mass.) at 2,000 rpm for 2 h. The partially purified viruses were then applied on to a 5 ml of 25% sucrose cushion for further purification. Sucrose gradient was centrifuged at 25,000 rpm for 2.5 h at 4°C in an SW55 rotor. Finally, the purified virus pellet was resuspended in Tris buffer (50 mM Tris-HCl [pH 7.4]). The resuspended virus was divided into aliquots, snap-frozen, and stored at -80°C. The titer of the purified virus preparation was determined by plaque assay on BHK cells and was found to be approximately 5 × 10^8 ^PFU/ml.

### Antibodies, reagents and chemicals

Mouse monoclonal antibody against DENV E protein was purchased from US Biologicals for immunofluorescence detection of DENV infection. Polyclonal antibodies specific for cellular proteins, clathrin and early endosomal antigen 1 (EEA1) were purchased from BD Pharmingen for immunofluorescence assays. The secondary antibodies conjugated to Texas red (TR) or fluorescein isothiocyanate (FITC) were purchased from Amersham Pharmacia Biotech. Lysotracker (a stain for late endosomes and lysosomes) was purchased from Molecular Probes. Monoclonal antibodies specific for the cellular proteins, CLTC (Sigma Aldrich), AP2B1 (Abcam), DNM2 (Sigma Aldrich), ARRB1 (Abnova), ATP6V0A1 (Sigma Aldrich) and ARPC1B (Santa Cruz Biotechnology) were used for Western detection. Chlorpromazine, filipin, nocodazole, cytochalasin D and bafilomycin A were purchased from Sigma Aldrich. All chemicals were ultragrade unless stated otherwise.

### siRNA library

The human genome siRNA subset library targeting the endocytic and membrane-trafficking genes (Dharmacon, RTF H-005500) was used in this study. A smart pool approach of incorporating four siRNAs targeting each gene was utilized. The advantages of this pooled approach as well as the issues of gene compensation of specific isotype of gene are discussed in [65]. The list of 119 targeted human genes and isoforms (excluding the control set) are given in Additional File 1.

### Reverse transfection of siRNA delivery into cells

All transfections were performed in a 384-well plate format. A 1.2% (vol/vol) stock solution of the transfection reagent (DharmaFECT 4) was prepared in DCCR cell culture buffer (Dharmacon) and incubated at 25°C for 10 min. From this stock, 8 μl was added to the lyophilized siRNA in each well of the 384 well-plate and incubated at 25°C for 30 min to allow the siRNAs to rehydrate and form siRNA-lipid complexes. Subsequently, 5 × 10^3 ^Huh7 cells in 42 μl of complete DMEM supplemented with 10% fetal bovine serum was added and the medium was changed after 24 h. The final concentration of pooled siRNAs per well was 50 nM. Individual siRNA duplexes were used at 6.25 pmol per well.

### Screening of the siRNA library

For the screening assay, a high-throughput platform for the specific detection of DENV infection in the 384-well plate format via immunofluorescence staining was described in [26]. In brief, the siRNA transfected cells were incubated at 37°C for 48 h (to ensure effective gene knockdown by the siRNA) before subjecting to DENV infection at an MOI of 1. After 48 h p.i., the cells were then fixed with cold absolute methanol (Sigma) per well in the 384-well format for 15 min at -20°C. The subsequent cell washing steps were carried out using an automated 384-well format plate washer (EMBLA, Molecular Devices). The cells were the subjected to immunofluorescence staining using the primary anti-DENV2 virus envelope antibody (US Biologicals) and followed by the secondary antibody conjugated with FITC (Millipore). The cell nuclei were then counter-stained with DAPI (100 nM; Molecular Probes) prior to collection of image data by the ArrayScan V^TI ^HCS automated fluorescence microscope Reader system (Cellomics) with the excitation wavelength (495 nm) and emission wavelength (520 nm) for FITC and the excitation wavelength (358 nm) and emission wavelength (461 nm) for DAPI. Data collection and auto-focusing parameters were preset using Cellomics Target Activation Bioapplication (Cellomics). A generic segmentation tool function was used to identify the two different stains (DAPI and FITC) with intensities above background staining and data collection was obtained by logging the measurements. Data analysis after image acquisition was carried out using the Cellomics Target Activation Bioapplication (Cellomics). DAPI-stained nuclei were counted to determine the total cell populations while FITC-stained cytoplasm was scored to determine the number of virus-infected cells. Any images with less than 500 cells were excluded from data analysis by the cell sorting module. Three independent screening assays were carried out.

The controls included in each individual set of experiments were the use of transfection reagent (DharmaFECT 4) alone, a non-targeting siRNA (Dharmacon), a green fluorescent non-specific siRNA (siGLO, Dharmacon), RISC-free siRNA (Dharmacon), and siRNA smart pools targeting Cyclo-B duplex, glyceraldehyde-3-phosphate dehydrogenase and lamin A/C and (Dharmacon). Furthermore, we have included the siRNA against *kif11 *gene which is required for cell survival, and siRNAs targeting this gene are cytotoxic. Therefore, *kif11 *siRNA provided a measure of siRNA efficacy with direct measurement of cell death. The other siRNAs served as negative controls for non-specific effects of siRNA and/or the transfection reagents on cell viability and virus infection. In addition, cell viability was checked by visually inspecting cells using phase-contrast microscopy.

### Dominant negative constructs of endocytic-trafficking mediators

Plasmid constructs of dominant-negative EPS15 (GFP-EPSΔ95/295, a component of the AP2 clathrin adapter complex; the dominant-negative form inhibits clathrin-coated pit budding), Rab5 S34N [a dominant-negative form that inhibits early endosome formation and trafficking, [40]] and the plasmid constructs backbone EGFP-C2 (Clontech) were transfected into Huh 7 cells and virus entry was then assessed. Unless stated otherwise, transfections were performed by using Lipofectamine LTX reagents from Invitrogen as specified by the manufacturer. In brief, Huh 7 cells were grown on coverslips in 24-well tissue culture plate until 60% confluency. Then, 3 μg portions of the respective plasmid constructs was complexed with 4 μl of Lipofectamine LTX reagent in 25 μl of OPTI-MEM medium (Gibco) for 15 min at room temperature. The mixture was then added to 25 μl of OPTI-MEM containing 2 μl of Lipofectamine. After incubation for another 15 min, the DNA-liposome complexes were added to the cells. After incubation for 3 h at 37°C, 1 ml of complete growth medium was added and incubated for another 24 h before virus entry assay was carried out.

### Indirect immunofluorescence and confocal microscopy

For immunofluorescence microscopy, cell monolayers were first grown on coverslips till 75% confluency. The subsequent procedure is similar to that described in reference [25]. The primary antibodies to clathrin and EEA1 were used at concentrations of 0.2 μg/ml and 0.1 μg/ml for anti-DENV antibody. Lysotracker was used at a concentration of 0.8 μM to stain specifically for late endosomes within cells. Secondary antibodies conjugated with either TR or FITC were used at a concentration of 0.1 μg/ml. The specimens were then viewed with a laser scanning confocal inverted microscope (Nikon A1R system) with an excitation wavelength of 543 nm for TR and 480 nm for FITC by using a 63× objective lens.

### Virus entry assay and drug treatments

Huh 7 cells growing on coverslips were incubated with DENV at an MOI of 1 for 1 h at 4°C with gentle rocking. Unbound virus was washed three times in ice-cold PBS and shifted to 37°C for 1 h in growth medium to allow virus penetration. Extracellular virus that failed to enter into cells was inactivated with acid glycine buffer (pH 2.8). Infected cell monolayers were washed twice with PBS and further incubated at 37°C for 48 h. At Day 2 p.i., cells were fixed in methanol and processed for immunofluorescence assay as described above. The number of infected cells is scored in comparison to mock-infected cells.

To determine the effects of the drugs used to inhibit the entry of DENV, Huh 7 cells were pretreated with drugs at different concentrations (as listed below) for 2 h at 37°C and followed by DENV infection. Cells were infected as described above and processed for immunofluorescence assay. Three independent experiments were carried out for each set of drugs used. The inhibition of virus entry was determined by determining the number of virus antigen-positive cells in relation to the total number of cells (virus antigen positive and negative) and is expressed as the percentage virus antigen-positive cells.

The drugs used in the present study were as follows: inhibitors of clathrin function, chlorpromazine (1 to 15 μg/ml); inhibitor of caveola-dependent endocytosis, filipin (0.1 to 2 μg/ml); vacuolar-ATPase specific inhibitor, bafilomycin A1 (0.01 to 1 μM); and inhibitor of the endocytotic trafficking pathway, cytochalasin D (0.1 to 2 μg/ml) and nocodazole (1 to 20 μM).

### Cytotoxicity Assays

The cytotoxicity of each of the small molecule inhibitors used in this study was assessed by incubating Huh7 cells with the different concentration range in culture medium in 96-well plates, corresponding to the incubation period of cells with compounds in the drug treatment assay. Following this, cell viability was assessed by 3- [4,5-dimethylthiazol-2-yl]-2,5-diphenyltetrazolium bromide (MTT assay, Chemicon, Temecula, CA) according to the manufacturer's recommendations.

## Competing interests

The authors declare that they have no competing interests.

## Authors' contributions

JJHC designed research; FA, APYW and JJHC performed research; FA, APYW, MLN and JJHC analyzed data and wrote the paper. All authors read and approved the final manuscript.

## Supplementary Material

Additional file 1 **Summary of human genes that are necessary for DENV infection**. The 119 siRNA of the targeted human genes and the brief description of the reported functional role for each of the genes are indicated in the table. Click here for file

Additional file 2 **Summary of human genes that are necessary for DENV infection**. The human genes that are required for the infectious entry of DENV are indicated in the table. Click here for file

Additional file 3 **Confocal microscopy 3D spectral reconstructed image of Huh7 cells infected with DENV**. Obvious co-localization of DENV with clathrin molecules are observed in yellow within the virus-infected cell. Click here for file
