# Supplementary material for: Small interference RNA profiling reveals the essential role of human membrane trafficking genes in mediating the infectious entry of dengue virus
Source: Virol J. 2010 Feb 1;7:24. doi: 10.1186/1743-422X-7-24 (PMC2825209; doi:10.1186/1743-422X-7-24)
Supplement: Additional file 1 — Summary of human genes that are necessary for DENV infection. The 119 siRNA of the targeted human genes and the brief description of the reported functional role for each of the genes are indicated in the table. [file 1743-422X-7-24-S1.DOC]

|  | |  |
| --- | --- | --- |
| **Gene Symbol** | **NCBI Gene Accession No.** | **Gene Name and Functional Roles** |
| ACTR2 | NM_005722 | ACTIN-RELATED PROTEIN 2; component ofArp2/3 complex promotes actin assembly in lamellipodia and may participate in lamellipodial protrusion |
| ACTR3 | NM_005721 | ACTIN-RELATED PROTEIN 3; component of Arp2/3 protein complex see ACTR2 |
| ADAM10 | NM_001110 | A DISINTEGRIN AND METALLOPROTEINASE DOMAIN 10 cell surface proteins with a unique structure possessing both potential adhesion and protease function possesses Tumor necrosis factor-alpha (TNFA) convertase activity |
| AMPH | NM_001635 | AMPHIPHYSIN synaptic vesicle-associated protein for mediating clathrin-mediated endocytosis |
| AP1B1 | NM_001127 | ADAPTOR-RELATED PROTEIN COMPLEX 1, BETA-1 SUBUNIT essential for the formation of adaptor complexes of clathrin-coded vesicles |
| AP1M1 | NM_032493 | ADAPTOR-RELATED PROTEIN COMPLEX 1, MU-1 SUBUNIT Heterotetrameric adaptor complexes promote the formation of clathrin-coated pits and vesicles |
| AP1M2 | NM_005498 | ADAPTOR-RELATED PROTEIN COMPLEX 1, MU-2 SUBUNIT member of the medium chain family of the clathrin-associated adaptor complex AP1 |
| AP2A1 | NM_014203 | ADAPTOR-RELATED PROTEIN COMPLEX 2, ALPHA-1 SUBUNIT alpha subunit is part of the so-called AP2 coat assembly protein complex which links clathrin to receptors in the coated vesicles |
| AP2A2 | NM_012305 | ADAPTOR-RELATED PROTEIN COMPLEX 2, ALPHA-2 SUBUNIT shares more than 97% sequence identity with rat alpha-adaptin |
| AP2B1 | NM_001282 | ADAPTOR-RELATED PROTEIN COMPLEX 2, BETA-1 SUBUNIT beta adaptin subunit of the clathrin coat assembly complex |
| AP2M1 | NM_004068 | ADAPTOR-RELATED PROTEIN COMPLEX 2, MU-1 SUBUNIT component of the AP2 coat assembly protein complex of clathrin-coated vesicles |
| ARF1 | NM_001658 | ADP-RIBOSYLATION FACTOR 1 involves in membrane trafficking, recruits and binds cytoplasmic COPI to the Golgi membranes constitute 1 family of the RAS superfamily |
| ARF6 | NM_001663 | ADP-RIBOSYLATION FACTOR 6 part of a group of Ras-like small guanosine triphosphatases (GTPases) called adenosine diphosphate (ADP)-ribosylation factors (ARFs) involve in intracellular membrane trafficking |
| ARFIP2 | NM_012402 | ADP-RIBOSYLATION FACTOR-INTERACTING PROTEIN 2 mediate crosstalk between RAC and ARF small GTPases; ARFs are implicated in vesicle transport between endoplasmic reticulum and the Golgi complex |
| ARPC1B | NM_005720 | ACTIN-RELATED PROTEIN 2/3 COMPLEX, SUBUNIT 1B part of the 7 subunits of Arp2/3 complex |
| ARPC2 | NM_005731 | ACTIN-RELATED PROTEIN 2/3 COMPLEX, SUBUNIT 2 |
| ARPC3 | NM_005719 | ACTIN-RELATED PROTEIN 2/3 COMPLEX, SUBUNIT 3 |
| ARPC4 | NM_005718 | ACTIN-RELATED PROTEIN 2/3 COMPLEX, SUBUNIT 4 |
| ARPC5 | NM_005717 | ACTIN-RELATED PROTEIN 2/3 COMPLEX, SUBUNIT 5 |
| ARRB1 | NM_004041 | ARRESTIN, BETA, 1, inhibits the signaling function of BARK-phosphorylated beta-adrenergic receptors Beta-arrestin-1 mutants, impaired either in SRC binding or in the ability to target receptors to clathrin-coated pits, acted as dominant negative inhibitors of beta-2 adrenergic receptor-mediated activation of the MAP kinases ERK1 and ERK2 |
| ARRB2 | NM_004313 | ARRESTIN, BETA, 2 regulate receptor coupling to G proteins;also function in internalization and signaling of these receptors |
| ATM | NM_138293 | ATAXIA-TELANGIECTASIA MUTATED GENE member of the phosphatidylinositol-3 kinase family of proteins that respond to DNA damage by phosphorylating key substrates involved in DNA repair and/or cell cycle control |
| ATP6V0A1 | NM_005177 | ATPase, H+ TRANSPORTING, LYSOSOMAL, V0 SUBUNIT A1, subunit of the vacuolar proton pump,ATP-driven proton pumps associated with the clathrin-coated vesicles and synaptic vesicles are a group of polypeptides involved in basic cellular processes through acidification of intracellular organelles. These functions include intracellular targeting of enzymes to lysosomes and secretory granules, and receptor-ligand dissociation in receptor-mediated endocytosis |
| BIN1 | NM_004305 | BRIDGING INTEGRATOR 1,features of a tumor suppressor protein, BIN1 bound specifically to integrins that are laminin receptors, including alpha-1, alpha-3A, alpha-3B, and alpha-6B |
| CAMK1 | NM_003656 | CALCIUM/CALMODULIN-DEPENDENT PROTEIN KINASE I;subfamily of the serine/threonine protein kinase family |
| CAV1 | NM_001753 | CAVEOLIN 1;main component of caveolae membranes |
| CAV2 | NM_001233 | CAVEOLIN 2;protein related to caveolin-1 ,similar in most respects;localizes to caveolae |
| CAV3 | NM_001234 | CAVEOLIN 3;muscle-specific form of the caveolin protein family |
| CBL | NM_005188 | CAS-BR-M MURINE ECOTROPIC RETROVIRAL TRANSFORMING SEQUENCE HOMOLOG functions as a negative regulator of several receptor protein tyrosine kinase signaling pathways and as an adaptor protein in tyrosine phosphorylation-dependent signaling |
| CBLB | NM_170662 | CAS-BR-M MURINE ECOTROPIC RETROVIRAL TRANSFORMING SEQUENCE B;high homology to CBL;negative regulator of autoimmunity |
| CBLC | NM_012116 | CAS-BR-M MURINE ECOTROPIC RETROVIRAL TRANSFORMING SEQUENCE C; phosphorylated upon activation of a variety of receptors that signal via protein tyrosine kinases, modulate downstream cell signaling |
| CDC42 | NM_001791 | CELL DIVISION CYCLE 42;GTP-binding protein; localized to the Golgi apparatus of mammalian cells. |
| CFL1 | NM_005507 | COFILIN 1;binds and depolymerizes filamentous F-actin and inhibits the polymerization of monomeric G-actin in a pH-dependent manner |
| CIB1 | NM_006384 | CALCIUM- AND INTEGRIN-BINDING PROTEIN 1;candidate regulatory molecule for integrin alpha-IIb-beta-3 |
| CIB2 | NM_006383 | CALCIUM- AND INTEGRIN-BINDING PROTEIN 2;play a role in the repair of double-strand DNA breaks and in the process of V(D)J recombination during lymphoid development |
| CIB3 | NM_054113 | CALCIUM- AND INTEGRIN-BINDING PROTEIN 3; homolog of CIB1 |
| CLTA | NM_001833 | CLATHRIN, LIGHT POLYPEPTIDE A;Two major classes of clathrin light chains, referred to as LCA (encoded by the CLTA gene) and LCB (encoded by CTLB) |
| CLTB | NM_001834 | CLATHRIN, LIGHT POLYPEPTIDE B |
| CLTC | NM_004859 | CLATHRIN, HEAVY POLYPEPTIDE;Clathrin molecules have a triskelion structure composed of 3 noncovalently bound heavy chains (CLTC) and 3 light chains |
| CLTCL1 | NM_001835 | CLATHRIN, HEAVY POLYPEPTIDE-LIKE 1 |
| DAB2 | NM_001343 | DISABLED, DROSOPHILA, HOMOLOG OF, 2; interacts with GRB2, an adaptor protein that couples tyrosine kinase receptors to SOS |
| DDEF2 | NM_003887 | DEVELOPMENT- AND DIFFERENTIATION-ENHANCING FACTOR 2;associated with GTPase activating protein (GAP) activity;DDEF2 is localized in the Golgi apparatus and at the plasma membrane, where it is colocalized with PYK2. DDEF2 forms a stable complex with PYK2 and activation of PYK2 leads to tyrosine phosphorylation of DDEF2 in vivo |
| DIAPH1 | NM_005219 | DIAPHANOUS, DROSOPHILA, HOMOLOG OF, 1;regulation of actin polymerization |
| DNM1 | NM_004408 | DYNAMIN 1;members of a subfamily of GTP-binding proteins that share considerable sequence homology in their N-terminal domains but more limited homology in their C-terminal regions.functions in receptor-mediated endocytosis and that it is required at an intermediate stage in coated vesicle formation;believed to assemble around the necks of clathrin-coated pits and assist in pinching vesicles from the plasma membrane |
| DNM2 | NM_004945 | DYNAMIN 2; DYN2 function as described for DNM1 and regulates actin reorganization at the immunologic synapse and links to VAV1 and its downstream signaling pathways after TCR engagement |
| DNM3 | NM_015569 | DYNAMIN 3; possess mechanochemical properties involved in actin-membrane processes, predominantly in membrane budding |
| EEA1 | NM_003566 | EARLY ENDOSOME ANTIGEN 1; marker for early endosomes and trafficking of endosomes |
| EFS | NM_005864 | EMBRYONAL FYN-ASSOCIATED SUBSTRATE |
| ELKS | NM_015064 | RAB6-INTERACTING PROTEIN 2; essential regulatory subunit of the IKK complex; Silencing ELKS expression by RNA interference blocked induced expression of NF-kappa-B target genes, including the NF-kappa-B inhibitor IKBA and proinflammatory genes such as cyclooxygenase-2 and interleukin-8 |
| ENTH | NM_014666 | EPSIN N-TERMINAL HOMOLOGY DOMAIN PROTEIN; accessory clathrin adaptors in endocytosis, Epsin is able to recruit and promote clathrin polymerisation on a lipid monolayer, but may have additional roles in signalling and actin regulation |
| EPN1 | NM_013333 | EPSIN 1;EPN1 is an endocytic accessory protein that interacts with EPS15, the alpha subunit of the clathrin adaptor AP2 (AP2A1), and clathrin, as well as with other accessory proteins for the endocytosis of clathrin-coated vesicles. |
| EPN2 | NM_148921 | EPSIN 2;NPF domain of EPN2 bound specifically to EPS15, while the DPW domain bound AP2 and clathrin |
| EPN3 | NM_017957 | EPSIN 3;The ENTH domain of EPN3 shares 80% and 82% sequence identity with those of EPN1 and EPN2, respectively, and the 3 NPF repeats are 100% conserved in these 3 proteins. EPN3 has a distal low-affinity clathrin-binding sequence in addition to the high-affinity site |
| EPS15 | NM_001981 | EPIDERMAL GROWTH FACTOR RECEPTOR PATHWAY SUBSTRATE-15;binds adaptor protein 2 |
| EPS15L1 | NM_021235 | EPIDERMAL GROWTH FACTOR RECEPTOR PATHWAY SUBSTRATE-15-LIKE 1 |
| FYN | NM_002037 | FYN ONCOGENE RELATED TO SRC, FGR, YES;member of the tyrosine kinase oncogene family; |
| GAF1 | NM_015470 | RAB11 binding protein |
| GIT1 | NM_014030 | G PROTEIN-COUPLED RECEPTOR KINASE-INTERACTING PROTEIN 1;cellular effects of Git1 required its intact ARFGAP activityThe paxillin-binding domain of GIT1 was required to recruit GIT1 to focal adhesions and the leading edge of the lamellipodia. The central ankyrin repeats and the PIX-binding domain were required to target GIT1 to the cytoplasmic complexes. Expression of GIT1 or its C-terminal paxillin-binding domain increased the rate of migration and the size and frequency of protrusions; PAK association was necessary for this effect. |
| GORASP1 | NM_031899 | GOLGI REASSEMBLY STACKING PROTEIN 1; interacts with GM130, a Golgi matrix protein, in detergent extracts of rat liver Golgi membranes;Interaction was also found to be critical for the correct targeting of both proteins to the Golgi apparatus. |
| GRB2 | NM_002086 | GROWTH FACTOR RECEPTOR-BOUND PROTEIN 2;mediating the growth factor-induced activation of RAS; implicated in growth factor regulation of the cytoskeleton and DNA synthesis |
| HIP1 | NM_005338 | HUNTINGTIN-INTERACTING PROTEIN 1;HIP1 is a membrane-associated protein that colocalizes with huntingtin and shares sequence homology and biochemical characteristics with Sla2p, a protein essential for function of the cytoskeleton in S. cerevisiae;endocytic protein, the structural integrity of which may be crucial for maintenance of normal vesicle size in vivo |
| HIP1R | NM_003959 | HUNTINGTIN-INTERACTING PROTEIN 1-RELATED PROTEIN;HIP1R share a conserved modular region termed the epsin N-terminal homology (ENTH) domain, which plays a crucial role in clathrin-mediated endocytosis;interaction between the ENTH domain and phosphatidylinositol-4,5-biphosphate is essential for endocytosis mediated by clathrin-coated pits |
| IHPK3 | NM_054111 | INOSITOL HEXAPHOSPHATE KINASE 3; IHPK3 converts InsP6 to InsP7 and might be involved in the regulation of vesicular dynamics |
| ITSN1 | NM_003024 | INTERSECTIN 1;Intersectin-1 is an evolutionarily conserved, multidomain protein that functions in clathrin-associated endocytosis and as a mediator of MAPK signaling pathways |
| ITSN2 | NM_006277 | INTERSECTIN 2; Overexpression of either of the ITSN2 isoforms or ITSN1 resulted in the inhibition of transferrin uptake and the blockage of clathrin-mediated endocytosis |
| LIMK1 | NM_002314 | LIM DOMAIN KINASE 1;acted in the nucleus to suppress Rac/Cdc42 signaling to cyclin D1;LIM kinase is phosphorylated and activated by ROCK, a downstream effector of Rho, and that LIM kinase, in turn, phosphorylates cofilin, inhibiting its actin-depolymerizing activity. |
| MAP4K2 | NM_004579 | MITOGEN-ACTIVATED PROTEIN KINASE KINASE KINASE KINASE 2; GC kinase specifically activates the SAPK pathhway |
| MAPK8IP1 | NM_005456 | MITOGEN-ACTIVATED PROTEIN KINASE 8-INTERACTING PROTEIN 1;C-terminal portions of JIP1 and JIP2 were sufficient for interaction with MLK3 and MKK7;JIP proteins function by aggregating components of a MAP kinase module, including MLK, MKK7, and JNK, and facilitate signal transmission by the protein kinase cascade |
| MAPK8IP2 | NM_012324 | MITOGEN-ACTIVATED PROTEIN KINASE 8-INTERACTING PROTEIN 2; see MAPK8IP1 |
| MAPK8IP3 | NM_015133 | MITOGEN-ACTIVATED PROTEIN KINASE 8-INTERACTING PROTEIN 3;JIP3 is a member of a novel class of putative MAPK scaffold proteins that may regulate signal transduction by the JNK pathway |
| NEDD4 | NM_006154 | NEURAL PRECURSOR CELL EXPRESSED, DEVELOPMENTALLY DOWNREGULATED 4; WW domains of NEDD4 bind with strong affinity to all 3 subunits of the epithelial sodium channel (ENaC): SCNN1A, SCNN1B, and SCNN1G. They concluded that both NEDD4 and the related gene KIAA0439 (NEDD4L) may play a role in the regulation of ENaC function. |
| NEDD4L | NM_015277 | UBIQUITIN PROTEIN LIGASE NEDD4-LIKE;By targeting the ENaC for degradation, NEDD4L is a significant determinant of sodium reabsorption in the distal nephron |
| NSF | NM_006178 | N-ETHYLMALEIMIDE-SENSITIVE FACTOR; member of the AAA (ATPases associated with diverse cellular activities) gene family involved in vesicular transport |
| PACSIN1 | NM_020804 | PROTEIN KINASE C AND CASEIN KINASE SUBSTRATE IN NEURONS 1;a family of cytoplasmic phosphoproteins that play a role in vesicle formation and transport. |
| PACSIN3 | NM_016223 | PROTEIN KINASE C AND CASEIN KINASE SUBSTRATE IN NEURONS 3;PACSINs are a family of cytoplasmic phosphoproteins that play a role in vesicle formation and transport |
| PAK1 | NM_002576 | p21/CDC42/RAC1-ACTIVATED KINASE 1; member of a family of serine/threonine kinases related to yeast Ste20 ;PAK is believed to act directly on the JNK1 MAP kinase pathway;MLCK is a target for PAK1, and that PAKs may regulate cytoskeletal dynamics by decreasing MLCK activity and myosin light-chain phosphorylation. |
| PICALM | NM_007166 | PHOSPHATIDYLINOSITOL-BINDING CLATHRIN ASSEMBLY PROTEIN;very high homology to the murine clathrin assembly protein ap3 |
| PIK3CG | NM_002649 | PHOSPHATIDYLINOSITOL 3-KINASE, CATALYTIC, GAMMA; PI3K plays a pivotal role in the regulation of cytotoxicity in NK cells |
| PIK4CA | NM_002650 | PHOSPHATIDYLINOSITOL 4-KINASE, CATALYTIC, ALPHA;catalyzes the first committed step in the biosynthesis of phosphatidylinositol 4,5-bisphosphate; protein is associated with the membranes of Golgi vesicles and vacuoles |
| PIP5K1A | NM_003557 | PHOSPHATIDYLINOSITOL-4-PHOSPHATE 5-KINASE, TYPE I, ALPHA; Phosphatidylinositol-4-phosphate 5-kinases (PIP5Ks) synthesize phosphatidylinositol 4,5-bisphosphate by phosphorylating phosphatidylinositol 4-phosphate. |
| PSCD3 | NM_004227 | PLECKSTRIN HOMOLOGY, SEC7, AND COILED-COIL DOMAINS PROTEIN 3; The PSCD proteins (PSCD1), a family of proteins containing a C-terminal pleckstrin homology (PH) domain and a central 200-amino acid region similar to a domain within the yeast Sec7 protein, which is required for vesicular traffic of polypeptides through the Golgi, function as guanine-nucleotide exchange factors (GEFs) for ARFs |
| RAB11A | NM_004663 | RAS-ASSOCIATED PROTEIN RAB11A;The Rab/Ypt proteins are the largest subgroup of the Ras superfamily and are differentially distributed in various cell types and intracellular compartments. |
| RAB11B | NM_004218 | RAS-ASSOCIATED PROTEIN RAB11B;The Rab family appears to play a critical role in regulating exocytotic and endocytotic pathways;RAB11B contains the 4 conserved domains important for GTP binding. The C terminus sequence of RAB11B is similar to that of RAB11A; in Rab proteins, this region is thought to be involved in membrane association |
| RAB3A | NM_002866 | RAS-ASSOCIATED PROTEIN RAB3A; interaction between mammalian synapsin I (SYN1) and Rab3a regulated the activities of both proteins. Synapsin I stimulated the Rab3a cycle by increasing GTP binding, GTPase activity, and Rab3a recruitment to the synaptic vesicle membrane. Conversely, Rab3a inhibited synapsin I binding to actin and synapsin I-induced synaptic vesicle clustering |
| RAB3B | NM_002867 | RAS-ASSOCIATED PROTEIN RAB3B; shares 78% identity with RAB3A ;Rab3b localized to vesicular structures containing Pigr in canine kidney cells and that GTP-bound Rab3b interacted directly with the cytoplasmic domain of Pigr. Binding of dIgA to Pigr dissociated Rab3b, but a constitutively active Rab3b mutant maintained its interaction with Pigr in dIgA-treated cells. Furthermore, GTP-locked Rab3b inhibited dIgA-stimulated transcytosis |
| RAB3C | NM_138453 | RAS-ASSOCIATED PROTEIN RAB3C; member RAS oncogene family; Protein transport. Probably involved in vesicular traffic (By similarity) |
| RAB3D | NM_004283 | RAS-ASSOCIATED PROTEIN RAB3D;highly expressed in YJ and HL-60 cells in response to retinoic acid treatment |
| RAB4A | NM_004578 | RAB4A, member RAS oncogene family ;Rab4 is involved in bidirectional sarcolemmal-vesicular Adrb2 trafficking |
| RAB4B | NM_016154 | RAB4B, member RAS oncogene family; Vesicular Protein transport. Probably involved in vesicular traffic (By similarity) |
| RAB5A | NM_004162 | RAS-ASSOCIATED PROTEIN RAB5A;RAB5 is a rate-limiting component of the machinery regulating the kinetics of membrane traffic in the early endocytic pathway. |
| RAB5B | NM_002868 | RAS-ASSOCIATED PROTEIN RAB5B;member of the GTPase superfamily RAB5B. It is presumably involved in vesicular trafficking at the plasma membrane |
| RAB5C | NM_004583 | RAS-ASSOCIATED PROTEIN RAB5C;shares 86% identity with RAB5A (179512) and RAB5B (179514) and contains the conserved GTP-binding site characteristic of RAS proteins |
| RAB6A | NM_002869 | RAS-ASSOCIATED PROTEIN RAB6A; Protein transport. Regulator of membrane traffic from the Golgi apparatus towards the endoplasmic reticulum (ER). Has a low GTPase activity |
| RAB6B | NM_016577 | RAB6B, member RAS oncogene family; Seems to have a role in retrograde membrane traffic at the level of the Golgi complex |
| RAB7L1 | NM_003929 | RAB7-LIKE 1; gene that shows sequence similarity to RAB7 and contains the 4 GTP-binding domains that are conserved in RAS proteins, although 2 of its domains have amino acid substitutions at conserved sites |
| RAB8A | NM_005370 | RAS-ASSOCIATED PROTEIN RAB8A; Rab8 is responsible for the localization of apical proteins in intestinal epithelial cells |
| RAB8B | NM_016530 | RAB8B, member RAS oncogene family; May be involved in vesicular trafficking and neurotransmitter release (By similarity) |
| RAC1 | NM_018890 | RAS-RELATED C3 BOTULINUM TOXIN SUBSTRATE 1; RAS superfamily involved actin polymerization |
| RHOA | NM_001664 | RAS HOMOLOG GENE FAMILY, MEMBER A;small guanosine triphosphatase (GTP) Rho regulates remodeling of the actin cytoskeleton during cell morphogenesis and motility. |
| ROCK1 | NM_005406 | RHO-ASSOCIATED COILED-COIL-CONTAINING PROTEIN KINASE 1; protein serine/threonine kinase that is activated when bound to the GTP-bound form of Rho |
| ROCK2 | NM_004850 | RHO-ASSOCIATED COILED-COIL-CONTAINING PROTEIN KINASE 2; ROCK2 is a serine/threonine kinase that regulates cytokinesis, smooth muscle contraction, the formation of actin stress fibers and focal adhesions, and the activation of the c-fos (164810) serum response element. ROCK2, which is an isozyme of ROCK1 (601702), is a target for the small GTPase Rho (e.g., 165390). |
| SH3GLB1 | NM_016009 | SH3 DOMAIN, GRB2-LIKE, ENDOPHILIN B1;knockdown of endophilin B1 in HeLa cells by RNA interference led to changes in mitochondrial shape, formation of vesicular structures continuous with mitochondria, and a separation of the outer and inner mitochondrial membrane compartments |
| SH3GLB2 | NM_020145 | SH3 DOMAIN, GRB2-LIKE, ENDOPHILIN B2; SH3GLB2 could form homodimers and heterodimers with SH3GLB1 in vitro |
| SNAP91 | NM_014841 | SYNAPTOSOMAL-ASSOCIATED PROTEIN; Snap91 is a synapse-associated protein; Snap91 is phosphorylated in vivo and that it is a substrate for Ca(2+)-dependent proteolysis |
| STAU | NM_004602 | STAUFEN, DROSOPHILA, HOMOLOG OF; STAU is localized to the rough endoplasmic reticulum;proposed that STAU plays a role in the targeting of RNA to its site of translation |
| SYNJ1 | NM_003895 | SYNAPTOJANIN 1; phosphatidylinositol (4,5)-bisphosphate (PI(4,5)P2) that has a role in clathrin-coated pit dynamics and can be recruited to clathrin-coated pits via a multiplicity of interactions |
| SYNJ2 | NM_003898 | SYNAPTOJANIN 2; regulating the formation of invadopodia and lamellipodia |
| SYT1 | NM_005639 | SYNAPTOTAGMIN I; integral membrane proteins of synaptic vesicles thought to serve as Ca(2+) sensors in the process of vesicular trafficking and exocytosis. Calcium binding to synaptotagmin I participates in triggering neurotransmitter release at the synapse;calcium binding to synaptotagmin I participates in triggering neurotransmitter release at the synapse |
| SYT2 | NM_177402 | SYNAPTOTAGMIN 2; integral membrane proteins of synaptic vesicles thought to serve as Ca(2+) sensors in the process of vesicular trafficking and exocytosis |
| TNIK | XM_039796 | TRAF2- AND NCK-INTERACTING KINASE;Germinal center kinases (GCKs), characterized by an N-terminal kinase domain and a C-terminal GCK domain that serves a regulatory function ; TNIK may be involved in cytoskeleton regulation;specific effector of RAP2 to regulate actin cytoskeleton |
| VAMP1 | NM_014231 | VESICLE-ASSOCIATED MEMBRANE PROTEIN 1;small integral membrane proteins specific for synaptic vesicles in neurons; |
| VAMP2 | NM_014232 | VESICLE-ASSOCIATED MEMBRANE PROTEIN 2; Synaptobrevin-2 is a small integral membrane protein specific for synaptic vesicles in neurons |
| VAPA | NM_003574 | VESICLE-ASSOCIATED MEMBRANE PROTEIN-ASSOCIATED PROTEIN A;VAPA interacts with VAMP1 and VAMP2 |
| VAPB | NM_004738 | VESICLE-ASSOCIATED MEMBRANE PROTEIN-ASSOCIATED PROTEIN B; member of the vesicle-associated membrane protein (VAMP)-associated protein (VAP) family. VAPB plays a role in the unfolded protein response (UPR), a process that suppresses the accumulation of unfolded proteins in the endoplasmic reticulum |
| VAV2 | NM_003371 | VAV2 ONCOGENE;an important role in cell signaling |
| VIL2 | NM_003379 | VILLIN 2; microvillar cytoplasmic peripheral membrane protein that is expressed strongly in placental syncytiotrophoblasts and in certain human tumors. It may contribute to setting the scaffold between the actin cytoskeleton and transmembrane proteins facilitating cell-cell interactions and receptor retention |
| WAS | NM_000377 | Wiskott-Aldrich syndrome; binds to and activates the Arp2/3 complex |
| WASF1 | NM_003931 | WISKOTT-ALDRICH SYNDROME PROTEIN FAMILY, MEMBER 1; The Wiskott-Aldrich syndrome protein (WASP), WASP-like (WASL), and WASF1 are among the downstream effector molecules involved in the transmission of signals from tyrosine kinase receptors and small GTPases to the actin cytoskeleton |
| WASF2 | NM_006990 | WISKOTT-ALDRICH SYNDROME PROTEIN FAMILY, MEMBER 2; WASF2 are among the downstream effector molecules involved in the transmission of signals from tyrosine kinase receptors and small GTPases to the actin cytoskeleton |
| WASF3 | NM_006646 | WISKOTT-ALDRICH SYNDROME PROTEIN FAMILY, MEMBER 3;WASF3 expression is strongest in brain;WASF3 binds actin through its C-terminal verprolin homology (VPH) domain. |
| PIK3C2G | NM_004570 | PHOSPHATIDYLINOSITOL 3-KINASE, CLASS 2, GAMMA; Phosphoinositide 3-kinases (PI3Ks) such as PIK3C2G regulate diverse cellular responses, such as cell proliferation, oncogenic transformation, cell migration, intracellular protein trafficking, and cell survival, by phosphorylating the hydroxyl group at the D-3 position of the inositol ring of phosphoinositides |
| MGC9726 | NM_177403 | RAB7B (RAB7B, member RAS oncogene family) |

**Additional File 1: The collection of genes and their functional roles within the siRNA membrane trafficking library.**
