# Supplementary material for: Small interference RNA profiling reveals the essential role of human membrane trafficking genes in mediating the infectious entry of dengue virus
Source: Virol J. 2010 Feb 1;7:24. doi: 10.1186/1743-422X-7-24 (PMC2825209; doi:10.1186/1743-422X-7-24)
Supplement: Additional file 2 — Summary of human genes that are necessary for DENV infection. The human genes that are required for the infectious entry of DENV are indicated in the table. [file 1743-422X-7-24-S2.DOC]

**Additional File 2: Summary of human genes that are required for DENV infection.**

| Gene and primary function | % of viral antigen positive cells | Reported function of gene product |
| --- | --- | --- |
| **Clathrin-coated pit formation** |  |  |
| AP1B1 | 46% | Subunit of clathrin-associated adaptor protein complex 1 |
| AP2A1 | 38% | Subunit of clathrin-associated adaptor protein complex 2 |
| AP2B1 | 33% | Subunit of clathrin-associated adaptor protein complex 2 |
| CLTB | 43% | Clathrin light polypeptide B; required for clathrin-coated pits |
| CLTC | 25% | Clathrin heavy polypeptide; required for clathrin-coated pits |
| CLTC1 | 39% | Clathrin heavy polypeptide-like 1; required for clathrin-coated pits |
| EPN1 | 39% | Epsin 1; endocytic accessory protein required for clathrin-coated pit formation |
| EPN2 | 33% | Epsin2; endocytic accessory protein required for clathrin-coated pit formation |
| EPS15 | 45% | Interacts with adaptor protein complex for formation of clathrin-coated pits |
| ITSN2 | 46% | Regulates formation of clathrin-coated vesicles |
| SYNJ1 | 39% | Recruited to clathrin coated pits and play functional role in clathrin coated pit dynamics |
| SYNJ2 | 41% | Recruited to clathrin coated pits and play functional role in clathrin coated pit dynamics |
| **Endocytosis** |  |  |
| ARRB1 | 39% | Receptor-mediated endocytosis in association with clathrin molecules |
| DNM1 | 36% | Regulates actin-membrane interaction for pinching off of endocytic vesicles from the plasma membrane |
| DNM2 | 26% | Regulates actin-membrane interaction for pinching off of endocytic vesicles from the plasma membrane |
| HIP1 | 44% | Requires for endocytosis and cytoskeleton rearrangment |
| HIP1R | 40% | Clathrin-mediated endocytosis |
| RAB11B | 32% | Associates with endocytic membrane component and play critical role in endocytosis |
| RAB3A | 41% | Associates with endocytic membrane component and play critical role in endocytosis |
| RAB3D | 43% | Associates with endocytic membrane component and play critical role in endocytosis |
| RAB5A | 38% | Associates with endocytic membrane component and play critical role in endocytosis |
| RAB7L1 | 39% | Associates with endocytic membrane component and play critical role in endocytosis |
|  |  |  |
| Gene and primary function | % of viral antigen positive cells | Reported function of gene product |
| **Signal transduction** |  |  |
| CAMK1 | 42% | Ca^2+^/calmodulin-dependent protein kinase involve in diverse signaling pathways |
| CBL | 43% | An adaptor protein in tyrosine phosphorylation-dependent signaling processes |
| CBLB | 39% | A homolog of CBL and is involved in tyrosine phosphorylation-dependent signaling processes |
| CBLC | 45% | A homolog of CBL and is involved in tyrosine phosphorylation-dependent signaling processes |
| CIB2 | 49% | Calcium- and integrin**-**binding protein 2 is involved in Ca^2+^ signaling processes |
| MAPK8IP2 | 39% | Involves in the c-Jun amino-terminal kinase signaling pathway |
| **Vesicle trafficking and maturation** |  |  |
| AMPH | 35% | Vesicle endocytosis and trafficking |
| ATP6V0A1 | 23% | Subunit of the vacuolar proton pump, ATP-driven proton pumps associated with the clathrin-coated vesicles and endosomes for the acidification processes |
| EEA1 | 28% | Early endosome antigen 1; early endosome maker and involved in trafficking of endosomes |
| ELKS | 37% | Intracellular vesicles trafficking from plasma membrane to Golgi apparatus |
| GORASP1 | 46% | Intracellular vesicles trafficking and correct targeting of proteins to the Golgi apparatus |
| PACSIN3 | 48% | Cytoplasmic phosphoprotein that plays a role in vesicle formation and transport |
| RAB8B | 44% | Vesicular endocytosis and trafficking |
| **Actin polymerization** |  |  |
| ACTR2 | 42 % | Component of actin related protein 2/3 complex implicated in actin assembly and polymerization |
| ARPC1B | 40% | Component of the actin related protein 2/3 complex (subunit 1B) implicated in actin polymerization |
| ARPC3 | 48% | Component of the actin related protein 2/3 complex (subunit 3) implicated in actin polymerization |
| DIAPH1 | 42% | Regulation of actin polymerization |
| PIP5K1A | 49% | Regulation of actin cytoskeletal remodeling |
| WASF2 | 45% | Involves in signaling through actin |
| **Others** |  |  |
| STAU | 48% | Plays a role in the targeting of RNA to its site of translation |
